# Magnetic Surface Molecularly Imprinted Polymer for Selective Adsorption of 4-Hydroxycoumarin

**DOI:** 10.3389/fchem.2022.862777

**Published:** 2022-04-07

**Authors:** Yi Kuang, Yunlong Xia, Xing Wang, Qingqing Rao, Shengxiang Yang

**Affiliations:** College of Chemical and Materials Engineering, Zhejiang A&F University, Zhejiang, China

**Keywords:** surface molecularly imprinted polymer, 4-hydroxycoumarin, selective adsorption, magnetic recycling, selective removal

## Abstract

4-hydroxyl coumarin (HC), an important intermediate during the synthesis procedure of rodenticide and anti-cardiovascular drug, shows highly medicinal value and economic value. To achieve the efficient adsorption of HC from natural biological samples, a novel magnetic surface molecularly imprinted polymer (HC/SMIPs) was constructed by employing methacrylic acid (MAA) as functional monomer, organic silane modified magnetic particles as matrix carrier and HC as template molecule. Due to the numerous specific imprinted cavities on the HC/SMIPs, the maximum adsorption capacity of HC/SMIPs for 4-hydroxycoumarin could reach to 22.78 mg g^−1^ within 20 min. In addition, HC/SMIPs exhibited highly selective adsorption for 4-hydroxycoumarin compared with other active drug molecules (osthole and rutin) and showed excellent regeneration performance. After 8 cycles of adsorption-desorption tests, the adsorption capacity of HC/SMIPs just slightly decreased by 6.64%. The efficient selective removal and easy recycle of 4-hydroxycoumarin from biological samples by HC/SMIPs made a highly promising to advance the application of imprinting polymers in complex practical environments.

## 1 Introduction

Coumarin, as a special natural product, are highly desirable for the widespread applications in biomedical systems and chemistry field due to the prominent pharmacological activities, such as anti-cancer (Zhao et al., 2019), antibacterial (Elias et al., 2019), anti-inflammatory (Yang et al., 2019), antioxidant (Mangasuli et al., 2019) etc. Coumarin is usually an endolipid compound with benzopyranone skeleton. Among them, 4-hydroxycoumarin is the key intermediate during the preparation procedure of anticoagulant and rodenticides, showing high medicinal value. Traditional adsorption by using adsorbents, such as activated carbon, molecular sieves and silica gel, is implementable and low cost (Fang et al., 2021; Yuan et al., 2018). However, the selectively adsorbing for target molecules is scarce. Hence, the development of novel adsorption method with high efficiency and selectivity to HC is extremely significant.

Recently, molecular imprinted technology (MIT) (Chen et al., 2016; Xu et al., 2021; Zhang 2020), as a rising molecular recognition technology, provides a new method to overcome the mentioned problems. In MIT, a specific target molecule or a structural analogue (virtual template) was firstly employed to facilitate recognition site formation through covalent or non-covalent interaction with bulk phase by polymerization, after removing the template, a molecularly imprinted polymer (MIP) was subsequent achieved with imprinted cavities which highly match the three-dimensional shape, size and functional site of target molecule (Gao et al., 2020; Pan et al., 2018; Si et al., 2018). Compared to other adsorption systems, MIPs exhibit three unique characteristics of structure predetermination, application universality and recognition specificity and are highly promising for solid phase extraction (Zhang et al., 2020), chemical sensors (Cheng et al., 2017; Lopez et al., 2021; Raziq et al., 2021), capillary electrophoresis (Bezdekova et al., 2021), simulated antibodies (Seitz et al., 2021) and chromatographic separation (Cheng et al., 2014). However, some drawbacks were founded in traditional molecular imprinting technology with the deepening development, including the serious embedding and uneven distribution of imprinting sites, incomplete removal of template, poor selectivity, and slow mass transfer rate, which directly limited the practical application of MIPs (Deng et al., 2012; Wulff 2002).

Nevertheless, surface molecular imprinting technology (SMIT) is capable of promoting the adsorption and recombination of imprinted molecules in a short time by limiting the imprinting sites onto the surface of the imprinted polymer (SMIP), which can avoid the above defects of MIT and meanwhile, significantly enhances the adsorption efficiency of SMIP (Tang et al., 2016; Zhou et al., 2022). During the fabrication of SMIP, selecting a suitable, efficient and stable matrix carrier is crucial (Li et al., 2020). So far, diverse inorganic materials have been developed as SMIP matrix carriers, such as SiO_2_, TiO_2_, Fe_3_O_4_ and various carbon nanoparticles due to their stable property, porous structure and large specific surface area (Wang et al., 2022; Wu et al., 2022; Zeng et al., 2022). For example, Liu et al. prepared a novel surface molecularly imprinted polymer (SMIP/MCNs) for specific adsorbing quinoline in coking wastewater by using the carbon nanospheres as the carrier (Cui et al., 2020), the adsorption capacity of SMIP/MCNs reached to 76.63 mg g^−1^ an initial quinoline concentration of 50 mg L^−1^. In addition, Luo and his coworker fabricated molecularly imprinted polymers (Fe_3_O_4_–MIPs) based on magnetic Fe_3_O_4_ nanoparticles for specific separation of protein (Yang et al., 2015). Fe_3_O_4_–MIPs exhibited high adsorption capacity and superb selective recognition toward targeted protein. Among these, the magnetic molecular imprinted polymers (M-MIPs) constructed with Fe_3_O_4_ particles can efficiently separate target molecules from mixtures by applying external magnetic field, that is conducive to recycling the adsorption materials with high efficiency and notably to implement compared with traditional separation and recovery technologies, such as centrifugation, filtration, etc (Huang et al., 2019; Li et al., 2019; Wu et al., 2017).

Herein, a novel magnetic surface molecularly imprinted polymer (HC/SMIPs) was fabricated for the specific extraction of 4-hydroxycoumarin based on silanized Fe_3_O_4_@SiO_2_ as the carrier, HC as the template molecule, methacrylic acid (MAA) as the functional monomer and ethylene glycol dimethacrylate (EGDMA) as the crosslinking agent through chemical modification and free radical polymerization. The microstructure and chemical composition of HC/SMIPs were investigated by scanning electron microscopy (SEM), X-ray diffraction (XRD), Fourier transform infrared (FT-IR) spectroscopy and thermogravimetric analysis (TGA). Notably, the abundant specific recognition sites on the HC/SMIPs surface greatly promotes the mass transfer efficiency and the selective recognition toward imprinted molecule. Specifically, the maximum adsorption capacity of HC/SMIPs could reach to 22.78 mg g^−1^ after 20 min. In addition, HC/SMIPs showed excellent selective recognition capacity to 4-hydroxycoumarin in ternary active substances mixture systems. The adsorption kinetics, adsorption isotherm, selectivity and regeneration performance of HC/SMIPs were studied in detail. The findings in this work could provide a theoretical basis for the application of surface molecular imprinted technology in the adsorption of 4-hydroxycoumarin.

## 2 Materials and Methods

### 2.1 Materials

3-methylacryloxy propyl trimethoxy silane (KH570) was purchased from Shandong Yusuo Chemical Technology Co., LTD. Tetraethyl orthosilicate (TEOS) was provided by Wuxi Yatai United Chemical Co., LTD. Ethylene dimethacrylate (EDGMA) was obtained from Shanghai Haiqu Chemical Co., LTD. Methacrylic acid (MAA) and 2,2′-Azobis (2-methylpropionitrile) (AIBN) were supplied by Tianjin Guangfu Fine Chemical Research Institute. 4-hydroxycoumarin (≥98%), rutin (≥98%), osthole (≥98%) were purchased from Shanghai Aladdin Chemical Reagent Co., Ltd. Ethanol was obtained from Hangzhou Qingchen Chemical Reagent Factory. Hydrochloric acid (HCl) was supplied by Chongqing Chuandong Chemical Co., LTD. All chemical reagents were of the best grade available and used as received. Deionized water was used throughout the whole experiments.

### 2.2 Fabrication of Magnetic Surface Molecularly Imprinted Polymer

#### 2.2.1 Fabrication of Fe_3_O_4_@SiO_2_

The Fe_3_O_4_ nanoparticles were prepared according to the previous reported method (Yuan, et al., 2016). In detail, FeCl_3_ (8.10 g), FeCl_2_.4H_2_O (6.60 g) and H_2_O (165.00 ml) were mixed with NH_3_·H_2_O (18.00 ml) and stirred vigorously for 5 min. Then the mixed solution was transferred to a 250 ml three-necked flask and continuously reacted at 60°C for 8 h. Subsequently, the obtained product was collected and repeatedly washed for 3 times with deionized water and ethanol respectively, and then dried at 25°C for 12 h to gain Fe_3_O_4_ nanospheres. Afterwards, Fe_3_O_4_ (0.10 g) was evenly dispersed in deionized water (30.00 ml) by ultrasound for 20 min and then poured into a three-necked flask. Whereafter, anhydrous ethanol (120.00 ml), concentrated ammonia water (3.00 ml) and TEOS (2.00 ml) were immediately added into the suspension and stirred at 30°C for 20 h. The prepared product was alternately washed with ethanol and deionized water for 3 times and further dried in a vacuum oven to obtain the Fe_3_O_4_@SiO_2_, in which the surface of Fe_3_O_4_ was covered with a layer of silica. Emphatically, the whole process was proceeded in nitrogen atmosphere.

#### 2.2.2 Fabrication of Silanized Fe_3_O_4_@SiO_2_ (KH570/Fe_3_O_4_@SiO_2_)

Firstly, HCl solution (4.00 mol/L, 100.00 ml) was mixed with Fe_3_O_4_@SiO_2_ (50.00 g) particles and standing for 24 h, then the prepared product was washed to neutral with deionized water and dried at 60°C for several hours to produce activated Fe_3_O_4_@SiO_2_. Secondly, KH-570 (3.00 ml), activated Fe_3_O_4_@SiO_2_ particles (10.00 g), ethanol (45.00 ml) and deionized water (15.00 ml) were added into a 100 ml three-necked flask, then the mixture was sonicated for 20 min and stirred magnetically at 65°C for 2 h. The prepared crude product was immediately washed with ethanol for several times and then dried at 60°C in a vacuum oven for 24 h, the purified product was named as KH570/Fe_3_O_4_@SiO_2_.

#### 2.2.3 Fabrication of Magnetic Surface Molecularly Imprinted Polymer

For a typical experiment, the HC (0.16 g) was firstly dissolved in the ethanol (50.00 ml) and then fully mixed with KH570/Fe_3_O_4_@SiO_2_ (0.50 g), MAA (0.56 ml), EGDMA (7.00 ml) and AIBN (0.15 g). Then, the mixed solution was injected into a 100 ml three-necked flask and stirred at 60°C for 6 h. Afterwards, the obtained matrix was repeatedly washed with ethanol to completely remove the template molecules HC, and then dried at 65°C for 12 h to produce the imprinted product, denoted as HC/SMIPs. Similarly, the surface non-imprinted polymer HC/SNIPs were fabricated under the same experimental procedure of HC/SMIPs without the addition of HC.

### 2.3 Characterizations

#### 2.3.1 Chemical Structure and Surface Topography

Fourier transform infrared (FT-IR) spectroscopy (FTIR-6700, Shimadzu Co., Ltd., Japan) was applied to test the chemical structure of samples. Thermal stability was recorded on a simultaneous thermal analyzer (TGA, STA449F3, Germany) in the air atmosphere and the heating rate was 10°C/min. The field emission scanning microscopy (SEM, Supra55, Germany) was employed to observe the surface morphology and the crystal structure of the samples was characterized by a TD-3500 X-ray diffractometer (XPS, China). In addition, magnetic properties of specimen were analyzed by a multi-functional vibrating sample magnetometer (FA200, Japan) at 293 K.

#### 2.3.2 Adsorption Experiments

##### 2.3.2.1 Adsorption Kinetics Test

Dynamic adsorption performance was tested by changing the adsorption time at intervals. Specifically, 11 groups of HC/SMIPs (40 mg) and were added to a conical flask containing the HC adsorption solution (4 ml, 300 μg mL^−1^), respectively. Then 1 ml supernatant of each sample was taken out and diluted to 10 ml by ethanol after binding for 3, 5, 10, 15, 20, 30, 40, 50, 60, 70 and 80 min, respectively. Afterwards, a UV-vis spectrophotometry (TU-1901, China) was employed to test the absorbance of the HC/ethanol solution before and after binding at the wavelength of 320 nm. The HC adsorption was than calculated according to the standard curve based on the change of absorbance. The adsorption capacity (Q) of the template molecule HC to the imprinted polymers is defined as:

$$\;Q=\left(C_{0}-C_{t}\right)V/W$$ (1)

where C_0_ and C_t_ refer to the concentration of HC at initial and time t, respectively. V is the volume of the adsorption solution, and W is the mass of the imprinted polymer. The adsorption kinetics experiment of HC/SNIPs was the same as that of HC/SMIPs. Each sample was performed at least three times in parallel, same with the following tests.

##### 2.3.2.2 Adsorption Isotherm Test

In order to investigate the adsorption capacity of HC/SMIPs, the experiment was carried out by changing the concentration of HC solution from 30 to 300 μg mL^−1^. Similarly, 9 groups of HC/SMIPs (10 mg) were added to HC solution with different concentration and then binding for 20 min. The adsorption capacity was then calculated as above.

##### 2.3.2.3 Selective Adsorption Test

Osthole and rutin were selected as comparative active substances to analyze the adsorption selectivity of HC/SMIPs. In detail, 3 groups of HC/SMIPs (40 mg) were severally added to a conical flask containing HC/ethanol, osthole/ethanol and rutin/ethanol solution (4 ml, 300 μg ml^−1^) and then binding for 20 min. The adsorption capacity was measured by UV-vis spectrophotometry at the wavelength of 320 nm for HC, 322 nm for Osthole and 278 nm for rutin and then calculated as described above.

##### 2.3.2.4 Competitive Adsorption Test

In order to investigate the selective recognition ability of imprinted polymers on template molecules in complicated environment, the adsorption of ternary active substances mixture based on HC, osthole and rutin with the concentrations of 300 μg/ml was carried out, ethanol was used as the solvent. HC/SMIPs or HC/SNIPs (40 mg) was added to the ternary mixed solution (12 ml) and binding for 20 min. The adsorption experiments and calculated method executed in the same way as mentioned above.

##### 2.3.2.5 Regeneration Test

The recyclability performance of HC/SMIPs were determined by the cyclic adsorption-desorption tests. The adsorption experiment was the same as described in adsorption kinetics test, the adsorption time of HC molecules and HC/SMIPs was set to 20 min. After adsorption, the imprinted polymers were collected by a magnet and washed several times with ethanol till the concentration of HC in the eluent is 0. Then the dried regenerated HC/SMIPs were used in the subsequent cycles of adsorption tests. Here, eight cycles of adsorption-desorption experiments were performed.

## 3 Results and Discussion

### 3.1 Preparation and Characterization of Magnetic Surface Molecularly Imprinted Polymer

**FIGURE 1:**
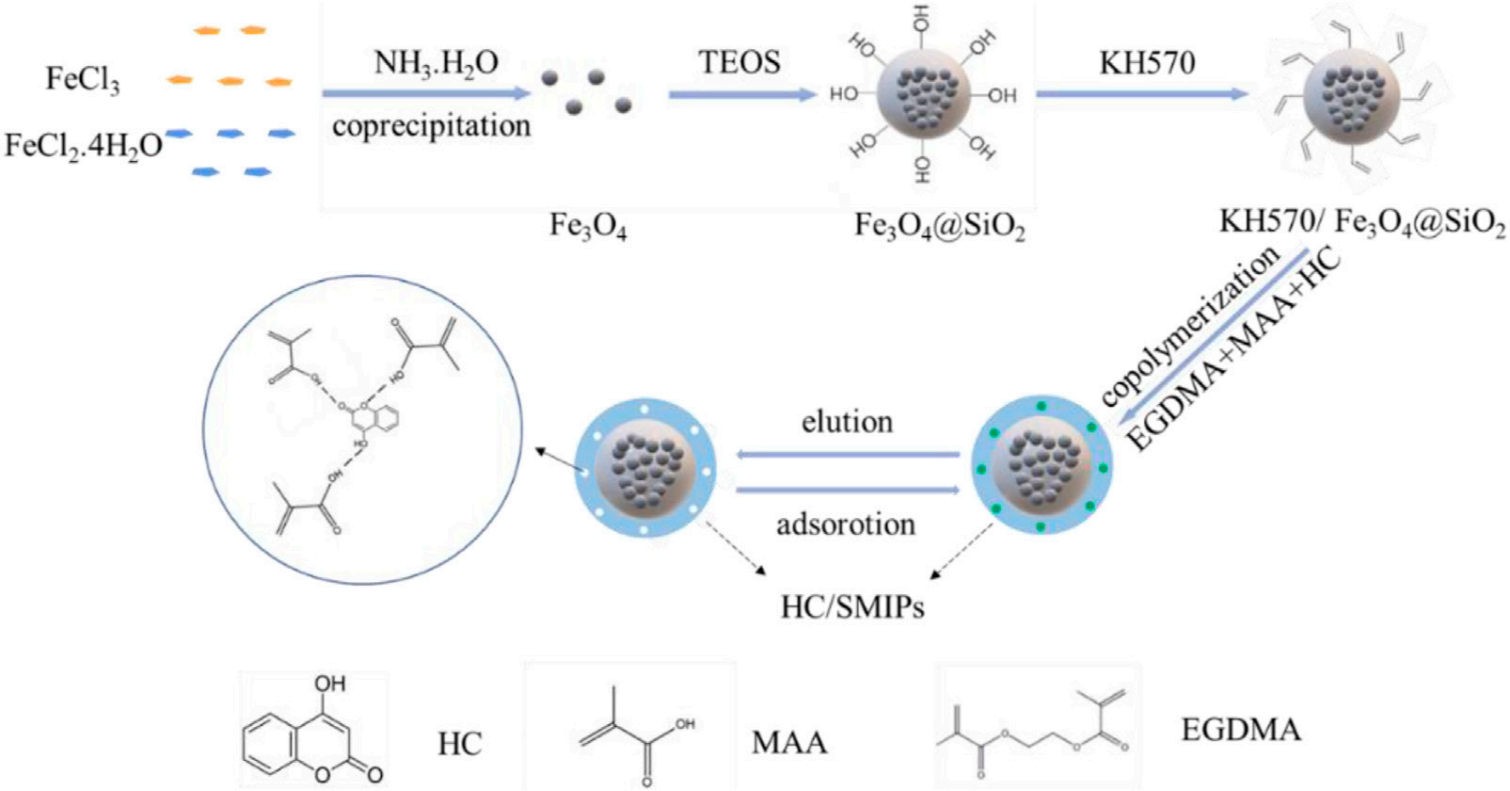
Schematic illustration of the preparation procedure of HC/SMIPs.

In a typical experiment, Fe_3_O_4_ nanoparticles were firstly prepared by chemical coprecipidation method, and then a layer of SiO_2_ was coated on the surface of magnetic particles through the hydrolysis condensation reaction of TEOS to produce Fe_3_O_4_@SiO_2_, as shown in Figure 1. These composite particles were further modified by silane coupling agent KH570 to introduce the C=C bonds on the surface. Afterwards, the template molecule was immobilized on the surface of imprinted polymers through the free-radical polymerization of MAA used as the functional monomer, EGDMA as the cross-linker and HC as the HC as the target matrix. Finally, the magnetic surface molecularly imprinted polymer with imprinted cavities complementary to HC in shape, size, and functional group orientation for adsorption of 4-hydroxycoumarin was obtained after elution of HC by polar solvent, named as HC/SMIPs.

**FIGURE 2:**
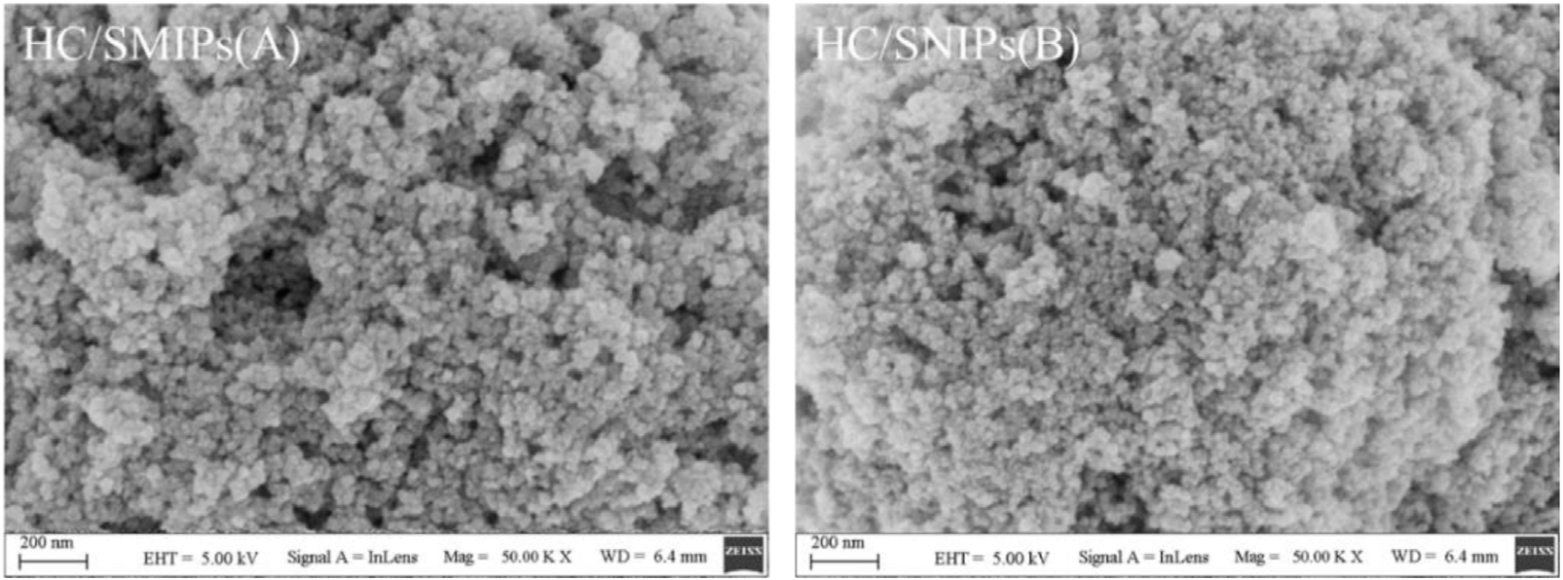
SEM images of the HC/SMIPs **(A)** and HC/NIPs **(B)**.

The micromorphology of HC/SMIPs and HC/SNIPs was characterized by SEM. As shown in Figure 2, the microspheres of HC/SMIPs and HC/SNIPs showed obvious agglomeration after covered with a layer of imprinting polymer, the process of polymerization and cross-linking around magnetic cores further promoted the agglomeration of indispersible magnetic particles. However, the surface roughness of HC/SMIPs is larger than that of HC/SNIPs prepared without template molecules, owing to the appearance of imprinted cavities after removing the imprinted molecules in HC/SMIPs. Then these imprinted cavities formed on the surface of imprinted layer could increase the adsorption area and meanwhile, promote the number of binding sites, thus improving the adsorption capacity of HC/SMIPs.

**FIGURE 3:**
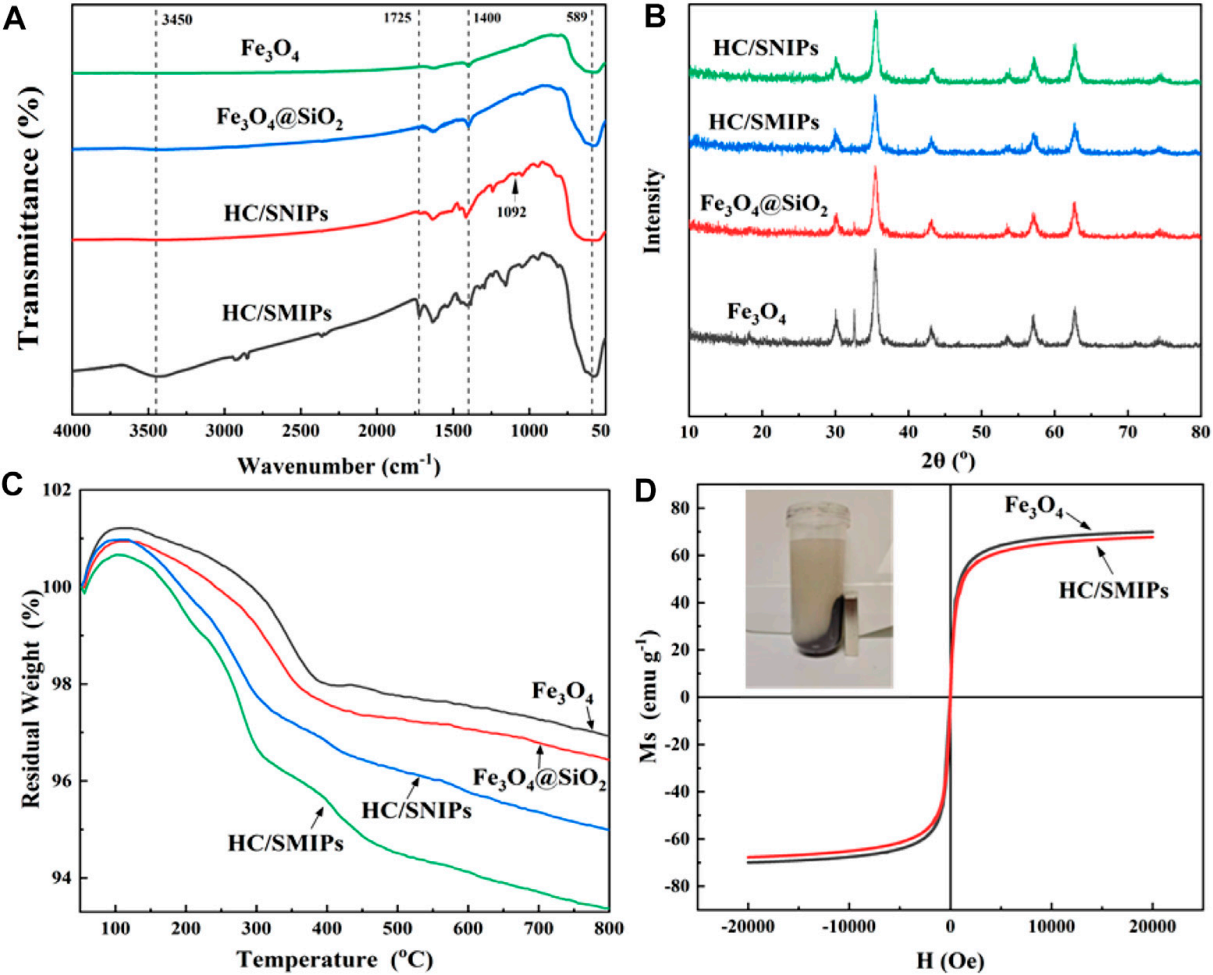
FTIR spectra **(A)**, XRD spectra **(B)** and TGA curves **(C)** of Fe_3_O_4_, Fe_3_O_4_@SiO_2_, HC/SNIPs and HC/SMIPs **(D)** Magnetic hysteresis loops of Fe_3_O_4_ and HC/SMIPs, the inset graph shows the magnetic separation of HC/SMIP in ethanol by a magnet.

Figure 3A showed the chemical composition of Fe_3_O_4_, Fe_3_O_4_@SiO_2_, HC/SNIPs and HC/SMIPs. The broad band at 589 cm^−1^ corresponded to the Fe-O stretching vibrations and the adsorption peak observed at 1092 was ascribed to the asymmetric vibrations of Si-O-Si. In addition, the stretching vibrations of C=O introduced by EGDMA was found around 1725cm^−1^ and the 3,450 cm^−1^ was assigned to the -OH adsorption peak. The absorption at 1400 cm^−1^ was mainly attributed to the C-H rocking bending vibrations of–CH_3_ and–CH_2_, and which was overlapped with the characteristic absorption peak of Fe-O bond. These results indicated the successful fabrication of imprinted polymer and the successful introduction of template molecule HC into HC/SMIPs. Notably, employing MAA as the functional monomer can not only improve specific recognition to imprinted molecules by the formation of hydrogen bonds with HC, but also can enhance the stability of imprinted cavity structure by constructing rigid network structure.

The crystal structures of Fe_3_O_4_, Fe_3_O_4_@SiO_2_, HC/SNIPs and HC/SMIPs were further analyzed by XRD (Figure 3B). As shown in the result, Fe_3_O_4_, Fe_3_O_4_@SiO_2_, HC/SNIPs and HC/SMIPs showed the same features. The obvious and sharp diffraction peaks were observed at 35°, 43°, 62°and 56°, respectively, which is adequately consistent with the standard PDF card for the crystal planes of Fe_3_O_4_ (JCPD card No. 88–0866). Except the slightly decrease of diffraction peak intensity in HC/SMIPs and HC/SNIPs, no obvious changes in the crystal structure were observed before and after imprinting. In addition, the thermal stability of above samples was shown in Figure 3C. As exhibited in the result, the mass of four samples all showed a slight increase trend in the initial stage possibly caused by the oxidation of Fe_3_O_4_. At the range of 50–800°C, the heat loss of Fe_3_O_4_ and Fe_3_O_4_@SiO_2_ was 3 and 4.5% for HC/SNIPs, 6% for HC/SMIPs, signifying the good thermal stability of HC/SMIPs and low covering rate of imprinted layer on the surface of carrier matrix.

To achieve the facilitate and efficient recovery of imprinted polymer, the magnetic Fe_3_O_4_ nanoparticles were employed as the carrier matrix to endow the imprinted system with magnetic responsiveness. As shown in Figure 3D, the magnetization curves of Fe_3_O_4_ and HC/SMIPs were all centrosymmetric with respect to the origin and in S-shape without hysteresis, indicating the excellent paramagnetic of Fe_3_O_4_ particles and HC/SMIPs. In detail, the magnetization saturation (Ms) values were 70.35 and 68.24 emu g^−1^ corresponded to the samples of Fe_3_O_4_ and HC/SMIPs, which were slightly decreased with the overlay of imprinted layer. The favorable magnetic performance of imprinted polymer could facilitate the separation of HC/SMIPs from ethanol by a magnet, as exhibited in the inset of Figure 3D, thus manifesting that HC/SMIPs can be used as an adsorbent to achieve efficient magnetic separation and recovery.

### 3.2 Adsorption Kinetics

**FIGURE 4:**
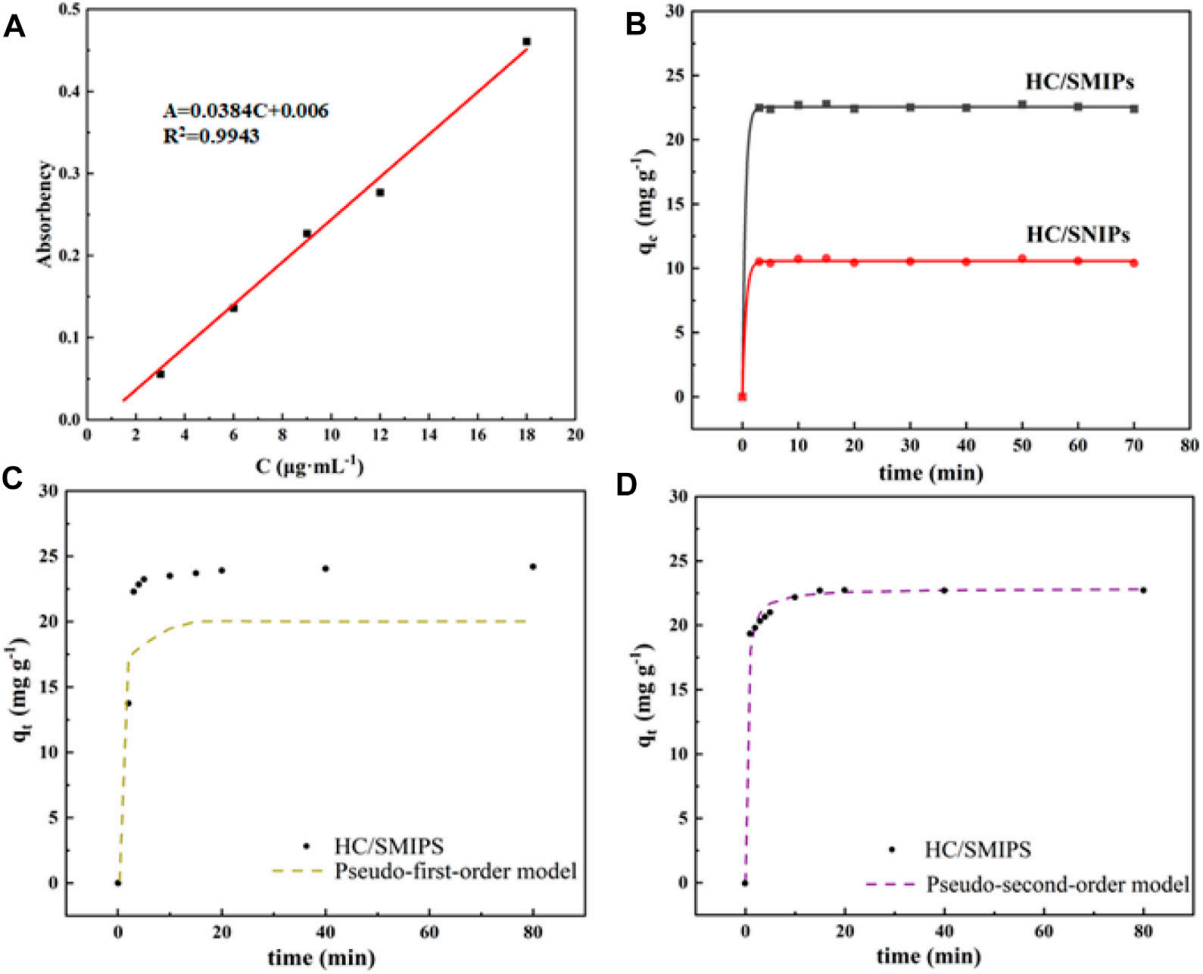
**(A)** Standard curves of HC adsorption **(B)** Adsorption kinetics curve of HC/SMIPs and HC/SNIPs; The Fitting adsorption curves of HC/SMIPs with pseudo-first-order kinetic model **(C)** and pseudo-second-order kinetic model **(D)**.

To investigate the saturated adsorption capacity and adsorption equilibrium time, also to reveal the internal adsorption behavior of HC/SMIPs, the adsorption kinetic of this magnetic adsorbent was measured. Figure 4A was the standard curve of HC adsorption. As shown in Figure 4B, the adsorption capacity of HC/SMIPs and HC/SNIPs on the template molecule HC increased rapidly with the increase of contacting time in the first 10 min. Then the adsorption capacity of these absorbents increased slowly until an adsorption equilibrium was achieved at 20 min. For an initial concentration of HC of 300 μg mL^−1^, the adsorption capacities of HC/SMIPs reached to 22.78 mg g^−1^, suggesting the HC/SMIPs had a relatively rapid adsorption rate and could quickly adsorb target molecule (Cheng et al., 2020). At the beginning of adsorbing process, numerous unoccupied recognition cavities were available on the surface of HC/SMIPs, meanwhile, the carboxyl groups on MAA could form hydrogen bonds with HC, so that the 4-hydroxycoumarin molecules were rapidly drawn to the surface of HC/SMIPs and be adsorbed with low resistance. Afterwards, most of imprinted cavities were gradually occupied and resulting in the higher diffusion resistance for targeted molecules to diffused into the residual cavities. Finally, all cavities were occupied and the adsorption equilibrium were achieved. However, the adsorption capacities of HC/SNIPs was only 10.79 mg g^−1^. The better adsorption performance of HC/SMIPs than that of HC/SNIPs was ascribed to the imprinted cavities on the surface of HC/SMIPs which could specifically match with HC. All these results clarified the good adsorption effect of HC/SMIPs.

Subsequently, pseudo-first-order kinetic model and pseudo-second-order kinetic model were used to fit the kinetic data to analyze the adsorption behavior and mechanism of HC/SMIPs on HC in ethanol solution. The corresponding adsorption kinetic equation were expressed as follows:

$$ln\left(Q_{e}-Q_{t}\right)=lnQ_{e}-k_{1}t$$ (2)

tQt=tQe+1k2Qe2 (3)

Where *Q*_*e*_ and *Q*_*t*_ refer to the adsorption capacity at adsorption equilibrium and time *t,* respectively. T is the adsorption time. K_1_ and K_2_ are rate constants of pseudo-first-order kinetic model and pseudo-second-order kinetic model, respectively.

The kinetic parameters of two kinetic models are listed in Table 1. As shown in the results, the adsorption behavior of HC/SMIPs for HC were more consistent with the pseudo-second-order kinetic model (Figures 4C,D). In detail, the correlation coefficient *R* ^2^ of pseudo-second-order model was 0.9999 and 0.9367 for pseudo-first-order model. In addition, the theoretical value of adsorption amount calculated by pseudo-second-order model was closer to the experimental data. The fitting results indicated that adsorption behavior of HC/SMIPs for HC was controlled by chemical adsorption and the adsorption capacity was proportional to the number of active binding sites in HC/SMIPs.

**TABLE 1:** Kinetic parameters for adsorption of HC on HC/SMIPs.

Adsorbent	q_e,exp_ (mg·g^−1^)	Pseudo-first-order			Pseudo-second-order		
		q_e,cal_ (mg·g^−1^)	k_1_ (min^−1^)	*R* ^2^	q_e,cal_ (mg·g^−1^)	k_2_ (mg·g^−1^·min^−1^)	*R* ^2^
HC/SMIPs	22.78	24.19	0.3402	0.9367	22.87	0.1608	0.9999

*q*_*e,exp*_ (mg·g^−1^) was measured by experiment; *q*_*e,cal*_ (mg·g^−1^) was calculated by fitting.

### 3.3 Adsorption Isotherm

To assess the binding ability and reveal the interaction between HC/SMIPs and 4-hydroxyl coumarin, the adsorption performance of HC/SMIPs and HC/SNIPs with various concentrations of template molecules at room temperature was investigated. The corresponding adsorption capacity equation were displayed as follows:

$$Q=\left(C_{0}-C\right)V/W$$ (4)

Where Q refers to the adsorption capacity. C and C_0_ are the concentration of 4-hydroxycoumarin at adsorption equilibrium and initial state. W is the mass of imprinted polymer and V is the volume of adsorption solution.

**FIGURE 5:**
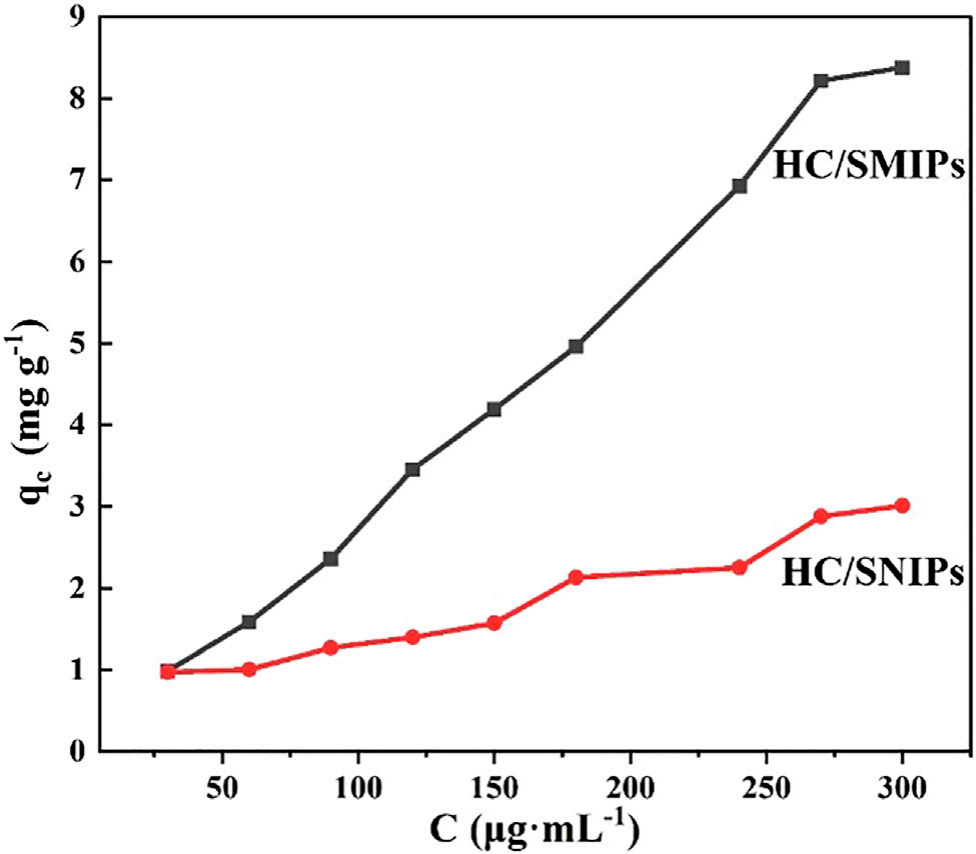
Adsorption isotherm curves for adsorption of HC on HC/SMIPs and HC/SNIPs.

As shown in Figure 5, both of the adsorption amounts of HC/SMIPs and HC/SNIPs were increased with the increase of the concentration of 4-hydroxycoumarin. Under the same adsorption conditions, the adsorption capacity of HC/SMIPs to HC was notably higher than that of HC/SNIPs and the adsorption rate of HC/SMIPs was also faster than that of HC/SNIPs, indicating the good specific recognition ability of HC/SMIPs to targeted molecule. The cavities in HC/SMIPs were highly matched with the size, shape and functional groups of template molecules, while HC/SNIPs had no specific recognition sites, Therefore the non-specific recognition of the target molecule in HC/SNIPs was dominant and the adsorption capacity was relatively low. In addition, the difference of HC/SMIPs and HC/SNIPs adsorption on HC gradually increased with the increase of the concentration of imprinted molecules. Hence, HC solution with the concentration of 300 μg mL^−1^ was employed for the following selective and competitive adsorption experiments.

### 3.4 Adsorption Selectivity

**FIGURE 6:**
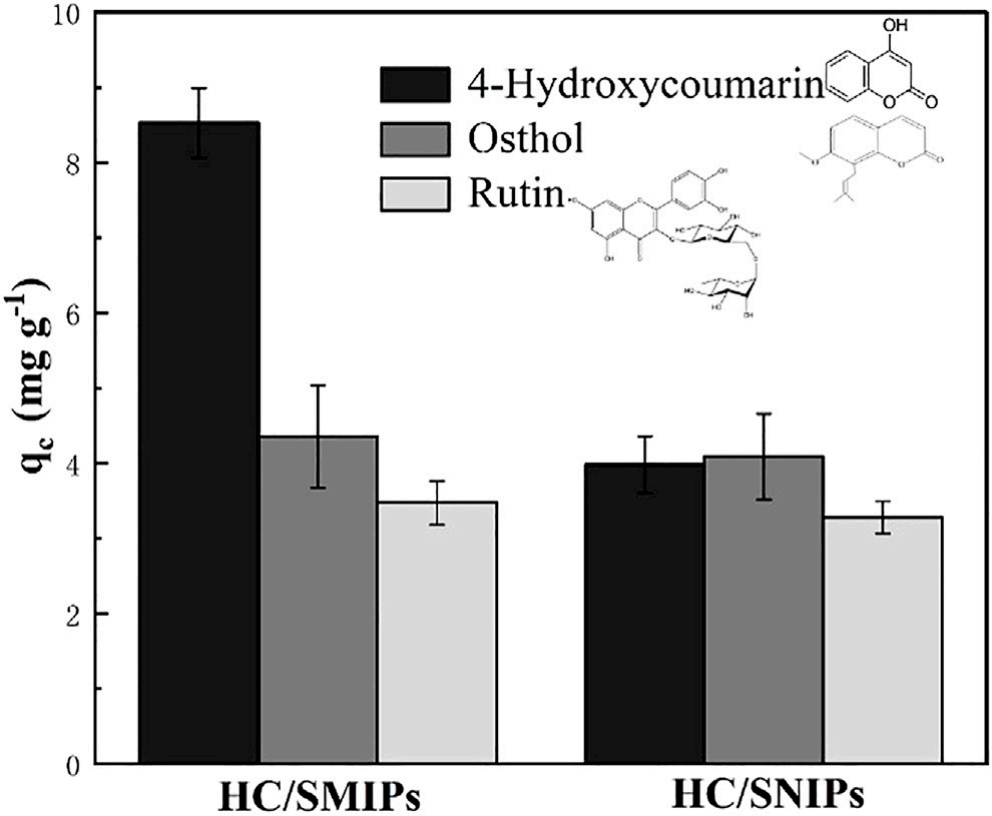
Selective adsorption of HC/SMIPs and HC/SNIPs.

In order to investigate the specific recognition of the HC/SMIPs for 4-hydroxycoumarin with the presence of other interferents, osthole and rutin with the similar structure of template molecule were selected as the competitive molecules to analyze the adsorption selectivity of HC/SMIPs. As shown in Figure 6, the adsorption amount of HC absorbed by HC/SMIPs was significantly higher than that for osthole and rutin, because HC/SMIPs possessed the better chemical affinity and steric matching with HC, rather than two other molecules. In addition, the adsorption capacities of HC/SNIPs on these three molecules were basically the same due to the lack of specific imprinted cavities. Moreover, the adsorption capacities of HC/SMIPs on osthole and rutin were similar to that of HC/SNIPs, showing a non-selective adsorption behavior. All these results demonstrated that HC/SMIPs had specific recognition ability to selectively bind HC.

### 3.5 Competitive Adsorption Property

The actual solution environment during adsorption process is very complicated and diversified. To better simulate the practical binding environment, ternary bioactive molecules mixture solution was applied to the competitive adsorption test, with HC as the template molecule, osthole and rutin as the competitive targets. The corresponding specific adsorption equation of the sample to the target molecule were described as follows:

$$K_{d}=Q/C$$ (5)

$$K=K_{dHC}/K_{dcompetitor}$$ (6)

$$K^{'}=K_{HC/SMIPs}/K_{HC/SNIPs}$$ (7)

Where *Q* is the adsorption quantity. *C* is the concentration of 4-hydroxycoumarin at adsorption equilibrium. *K*_*d*_ refers to the partition coefficient, which can be used to evaluate the binding ability of imprinted polymer to targeted molecule. *K* is selectivity coefficient and refers to the adsorption selectivity of imprinted materials for competitors. K′ is the relative selectivity coefficient and represents the difference between different imprinted polymers.

**FIGURE 7:**
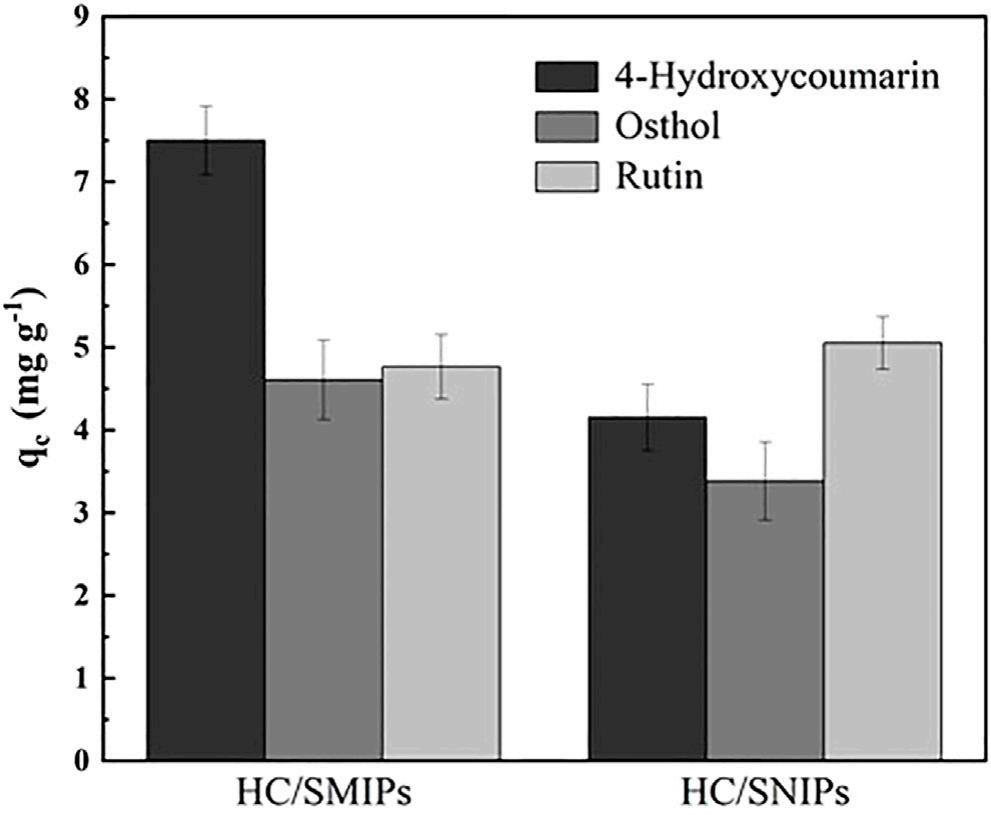
The specific binding amount of HC/SMIPs and HC/SNIPs for HC, osthol and rutin.

The selectivity parameters and competitive adsorption property were displayed in Table 2 and Figure 7. As shown in the results, the adsorption amount of HC/SMIPs for HC was significantly higher than that of osthole and rutin in the competitive adsorption environment, specifically, it was 1.62 times than the adsorption capacity for osthole and 1.57 times than the amount for rutin. In addition, the adsorption behavior of HC/SMIPs for two competitive analogues is similar to that of HC/SNIPs, demonstrating the non-specific adsorption of HC/SMIPs for osthole and rutin and terrifying the successful construction of imprinted cavities with good shape memory effect towards targeted molecule. HC/SNIPs showed no obvious discrimination among three bioactive molecules and the selectivity coefficients of HC/SNIPs for ostholes and rutin were 1.24 and 0.79, respectively, that was relatively low. All the results showed that HC/SMIPs has good specific adsorption performance and is highly promising to complicated environment for binding 4-hydroxycoumarin.

**TABLE 2:** The selectivity parameters of HC/SMIPs and HC/SNIPs.

	C (mg·mL^−1^)		Q (mg·g^−1^)		K_d_ (mL·g^−1^)		K		K′
	HC/SMIPs	HC/SNIPs	HC/SMIPs	HC/SNIPs	HC/SMIPs	HC/SNIPs	HC/SMIPs	HC/SNIPs	
HC	0.22	0.26	7.50	4.15	33.35	16.08	-	-	-
Osthol	0.25	0.26	4.61	3.38	18.15	12.93	1.84	1.24	1.48
Rutin	0.25	0.25	4.76	5.05	18.88	20.25	1.77	0.79	2.22

### 3.6 Regeneration Performance

**FIGURE 8:**
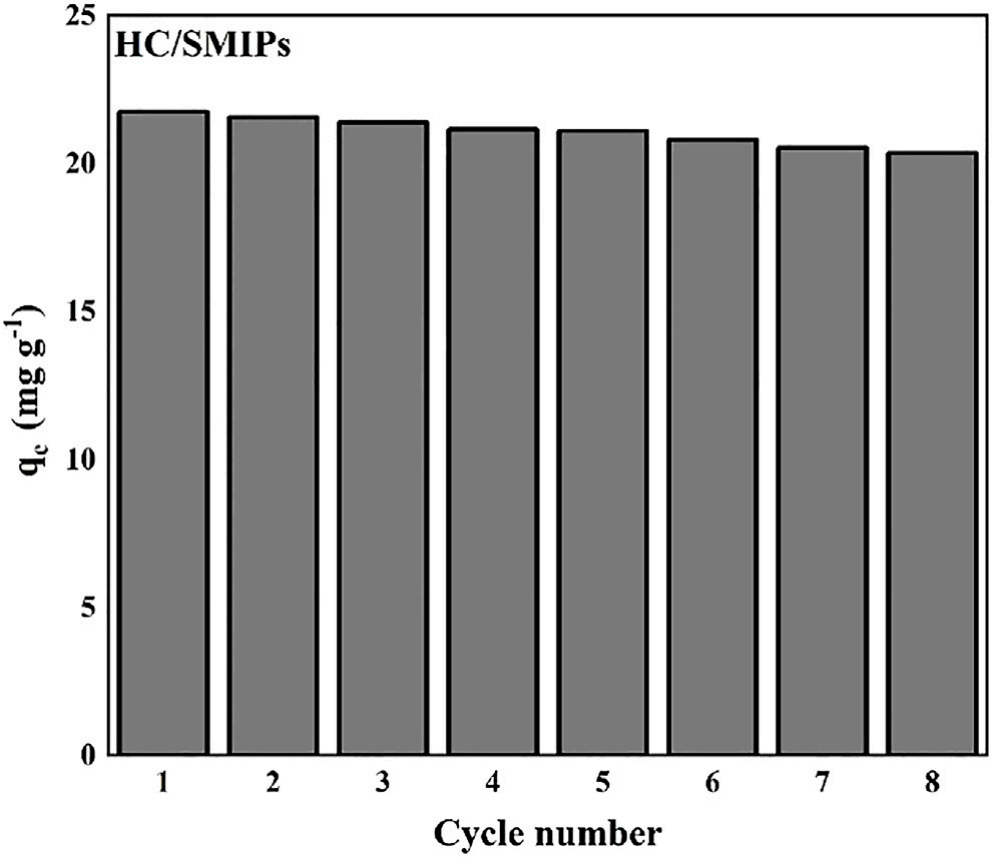
Regeneration performance of HC/SMIPs.

The recyclability performance was an important factor for adsorbents during the process of practical application. Here, the stability and regeneration properties of HC/SMIPs were determined by the cyclic adsorption-desorption tests. As shown in Figure 8, the adsorption efficiency of HC/SMIPs for 4-hydroxycoumarin showed no significant change. The adsorption capacity of HC/SMIPs was only decreased by 6.64% from 22.78 to 20.35 mg g^−1^ after 8 cycles, showing the good durability and regeneration performance of HC/SMIPs. On the one hand, a few of imprinted cavities were damaged by the repeated adsorption-desorption behavior; on the other hand, the incomplete elution of imprinted molecules resulted in a small amount of HC remaining in imprinted complex particles, therefore reducing the effective adsorption sites of absorbents and causing the slight decrease of adsorption capacity. In general, HC/SMIPs possessed good regeneration performance and the introduction of magnetic matrix could effectively simplify the separation process, so as to facilitate the regeneration of HC/SMIPs.

## 4 Conclusion

In this work, a novel magnetic surface molecularly imprinted polymer (HC/SMIPs) was fabricated by employing the 4-hydroxycoumarin as template molecule, silylated Fe_3_O_4_@SiO_2_ as the carrier matrix and MAA as the functional monomer. HC/SMIPs showed high adsorption capacity, fast adsorption kinetics and good specific recognition ability. The adsorption amount of HC/SMIPs for HC could reach to 22.78 mg g^−1^ within 20 min, meeting the demand of rapid adsorption rate as an absorbent. The adsorption kinetics behavior of imprinting polymer met the pseudo-second-order kinetic model. In addition, HC/SMIPs exhibited high selectivity for HC in ternary bioactive molecules mixture system and excellent regeneration, the adsorption capacity still maintained 20.35 mg g^−1^ after 8 cycles of adsorption-desorption tests. Moreover, the introduction of magnetic particles could further simplify the regeneration process. Therefore, HC/SMIPs prepared in this work could be a promising adsorbent for the specific removal and resource recycling of 4-hydroxycoumarin from practical biological samples.

## Data Availability

The original contributions presented in the study are included in the article/Supplementary Material, further inquiries can be directed to the corresponding authors.
